# Enhancing the referral process for surgical management of spinal metastases: insights from a 12-year, bi-institutional study of 533 patients

**DOI:** 10.3389/fonc.2024.1301305

**Published:** 2024-01-30

**Authors:** Bertrand Debono, Alexis Perez, Guillaume Lonjon, Olivier Hamel, Jean-Baptiste Dandine, Martin Dupuy, Guillaume Dutertre, Cécile Braticevic, Igor Latorzeff, Aymeric Amelot

**Affiliations:** ^1^ Department of Neurosurgery, Paris-Versailles Spine Center, Hôpital privé de Versailles, Versailles, France; ^2^ Department of Neurosurgery, Clinique de l’Union, Toulouse, France; ^3^ Department of Orthopedic Surgery, Orthosud, Clinique St-Jean-Sud de France, Santé Cite Group, Montpellier Metropole, France; ^4^ Department of Neurosurgery, Clinique des Cédres, Toulouse, France; ^5^ Institut Curie, Paris Sciences et Lettres (PSL) Research University, Surgical Oncology Department, Paris, France; ^6^ Department of Medical Oncology, Institut Paoli-Calmettes, Marseille, France; ^7^ Department of Radiotherapy, Groupe ONCORAD Garonne, Clinique Pasteur, Toulouse, France; ^8^ Department of Neurosurgery, Hopital Bretonneau, Tours, France

**Keywords:** spine metastasis, tumor board, referral, delay, emergency, spine surgery

## Abstract

**Introduction:**

Delayed surgical management of spinal metastases (SMs) can have detrimental effects on patient survival and quality of life, leading to pain and potential neurological impairment. This study aimed to assess the impact of delayed referral for SMs on clinical outcomes by analyzing patients managed in emergency situations.

**Methods:**

We retrospectively reviewed the data of all patients admitted on either emergency or elective basis who underwent surgery for the treatment of neoplastic spine lesions at our two institutions (tertiary referral neurosurgical units) between January 2008 and December 2019.

**Results:**

We analyzed 210 elective (EGp) and 323 emergency patients (UGp); emergencies increased significantly over the 12-year period, with a Friday peak (39.3%) and frequent neurological impairment (61.6% vs. 20%). Among the UGp patients, 186 (7.5%) had a previously monitored primitive cancer, including 102 (31.6%) with known SMs. On admission, 71 of the 102 (69.9%) patients presented with neurological deficits. UGp patients were more likely to undergo a single decompression without fixation. Outcomes at the 3-month follow-up were significantly worse for UGp patients ([very] poor, 29.2 vs. 13.8%), and the median overall survival for UGp patients was statistically lower. Risk factors for patients with SM undergoing emergency management included short delay between onset of symptoms and first contact with a spine surgeon, and an initial motor deficit.

**Conclusion:**

Many patients with previously identified metastases, including those with neurological deficits, are urgently referred. Optimization is needed in the oncology pathway, and all stakeholders must be made aware of the factors contributing to the improvement in the clinical and radiological identification of potential complications affecting patient survival and quality of life.

## 1 Introduction

The combination of aging populations, a better physio-pathological understanding, and improved treatment options is leading to an increase in the incidence of cancers (1, 2). Improved patient survival is associated with an increased incidence of metastases, that frequently involve the spine (3, 4). While some post-mortem studies have suggested that up to 70% of patients diagnosed with cancer will eventually develop spinal metastases (5), recent reviews highlighted that the clinical incidence of spinal metastases is approximately 15% (2/3 from a breast, prostate or lung neoplasia), of which up to 10% will develop metastatic spinal cord compression, and about 12% will suffer a metastatic vertebral compression fracture (6). The management of spinal metastasis (SM) requires optimal multidisciplinary cooperation, whether the primary cancer is synchronous or has already been identified (7). Prior to the 1990s, radiotherapy was the treatment of choice (8), until the paradigm shift triggered in 2005 by the study by Patchell et al., demonstrating that, regardless of the nature of the epidural compression, surgery followed by adjuvant conventional radiotherapy was considered superior to conventional radiotherapy alone, with preserved ambulatory function in 84% and 54% of cases, respectively (odds ratio [OR] 6.2, p=0.001), and significantly longer preservation of ambulatory function (median 122 and 13 days, respectively, p=0.003) (9).

Nevertheless, surgery poses major challenges, including pain control and ensuring biomechanical safety to avoid neurological complications (10, 11). Regardless of the circumstances of SM diagnosis, treatment delay is a major factor affecting patients’ quality of life and survival, especially when metastases are discovered in an emergency (12–14).

Several authors have highlighted dysfunctions that can delay the referral of these patients, including anomalies throughout the therapeutic pathway (15–17). Galasko et al. (18) and Portmans et al. (19) highlighted the unacceptable delay in the diagnosis and treatment of metastatic spinal cord compressions, stating that a lack of knowledge (particularly concerning spinal instability) was a reason for supply chain failure. Moreover, in 2013, the same team of authors highlighted a lack of progress across the cancer care pathway, resulting in treatment delays and the loss of patient opportunities (20).

Despite significant advances in knowledge and technology, organizational improvements, and massive public health investments in cancer care, we hypothesize that deficits remain in the referral of patients with SM. We aimed to analyze the context of referral and management of patients with SM by identifying persistent problems, developing a more general hypothesis concerning the care pathway and organization of upstream oncological management, and highlighting the necessary changes.

## 2 Materials and methods

### 2.1 Ethics

The data collected during the study were stored in a computer file in accordance with the law of the French Data Protection Act of January 6, 1978, amended in 2004. The protocol can be found in the reference methodology chapter MR003, adopted by the National Commission for Information Technology and Civil Liberties, which conforms to the standards of the institutions involved in this project. This study was approved by the institutional review board of the French College of Neurosurgery (August 2022; no. IRB00011687).

### 2.2 Patient series

This series included all patients who presented with SM at two tertiary neurosurgical centers, covering approximately 75% of the overall activity of all spinal surgery centers in our area (21), from January 2008 to December 2019.

In our prospectively acquired database, patients were dichotomized according to their mode of first contact with the spinal surgeon: emergency (urgent group, UGp) or elective consultation (elective referrals group, EGp). All patients surgically treated for SM with a known or unknown primary neoplasm, regardless of their neurological status, were included in the study. The exclusion criteria included primitive spine tumors and patients who did not undergo surgery.

### 2.3 Clinical and radiological characteristics

Prospectively acquired clinical records of patients were retrospectively analyzed. Age, sex, clinical signs, Karnofsky performance status (KPS), radiological findings, and histopathological results were systematically obtained from medical records. Neurological status was evaluated using the Frankel classification (22). We retrospectively evaluated patient data according to the revised Tokuhashi score (23). If the lesion was revealing or the patient suffered from a known neoplasm, we checked the length of evolution of the primitive disease and whether an SM had already been identified and the patient received a surgical proposal before referral to our institution.

### 2.4 Referrals

We analyzed the referrals and divided them into two groups: elective (i.e., referred to a scheduled consultation) and emergency (by urgent and direct contact with the on-call surgeon from a physician or corresponding institution from our region, including the internal or external emergency department).

### 2.5 Metastasis-free survival and clinical follow-up

MFS was defined as the period between the primary cancer diagnosis and SM diagnosis (24). Clinical outcomes were assessed based on symptoms at initial hospitalization (neurological deficit and pain) according to a Likert-like scale: very good (total improvement), good (significant improvement), fair (partial improvement), poor (minimal or no improvement), and very Poor (major deterioration or death).

### 2.6 Statistical analyses

All tests were two-sided, and statistical significance was set at p <0.05. Statistical analyses were conducted using SPSS software (version 22.0; SPSS, Chicago, IL, USA). Nomograms were established and verified using R version 3.2.5, with the Rms package (Design, Vienna, Austria). Data are presented as the mean ± standard deviation (SD). Sex and vertebral localization of metastases were considered categorical variables, whereas age and follow-up duration were considered continuous variables. Categorical variables were described as frequencies and percentages, whereas continuous and normally distributed variables were described as means and SDs. In the univariate analysis, categorical variables were assessed using Pearson’s chi-squared or Fisher’s exact test. Multivariate analysis was conducted separately for each diagnosis, and Cox proportional hazards models were used to estimate hazard ratios and 95% confidence intervals (CIs). The output is expressed as ORs and their bootstrapped 95% CIs. The Kaplan–Meier method was used to estimate MFS. For descriptive and inferential analyses, bootstrapping with replacement (iterations = 1000) was performed to obtain variance estimates at a 95% CI.

## 3 Results

### 3.1 Baseline characteristics

Our series consisted of 533 patients who underwent surgery for SM from 2008 to 2019, including 323 (60.6%) and 210 (39.4%) in the UGp and EGp, respectively (**Table 1**). More neurological deficits (61.6% vs. 20.0%, p<0.0001) with a non-ambulatory status (Frankel A–C: 32.5% vs. 2.6%, p<0.0001) were observed in the UGp. There was significantly more involvement of the thoracic level (68.4% vs. 46.2%, p<0.0001) and more intracanal epidural invasion (85.1% vs. 72.9%, p=0.001) in the UGp. The mean KPS score was lower in the UGp than in the EGp (KPS 76.2 vs. 86.1, p<0.0001). Primary tumor repartition was significantly different between the UGp and EGp (p<0.0001).

**Table 1:** Pre-treatment baseline characteristics for patients referred for spine metastases (2008–2019) according to the admission pathway (n=533).

	Emergency (n=323)	Elective (n=210)	p value
Demographics			
Mean age, years (SD)	65.1 (11.4)	65.9 (11.1)	.638
Gender, n (%) Male Female	211 (65.3) 112 (34.7)	103 (49.1) 107 (50.9)	**.001**
Addressing center, n (%) GP Oncology Emergency Room Another specialist	53 (16.4) 126 (39.0) 68 (21.1) 76 (23.5)	47 (22.4) 123 (58.6) 1 (0.5) 39 (18.6)	**.001**
Evolution before referrals Day Weeks Months	125 (38.7) 68 (21.0) 130 (40.2)	10 (4.7) 103 (49.0) 97 (46.2)	**<.0001**
Symptoms type Pain Neurologic	199 (61.6) 124 (38.4)	168 (80.0) 42 (20.0)	**.001**
Mean KPS (SD)	76.2 (13.9)	86.13 (7.0)	**.001**
Frankel on entry, n (%) A B C D E	4 (1.2) 27 (8.4) 78 (24.1) 96 (29.7) 122 (37.8)	0 (0) 1 (0.5) 5 (2.4) 36 (17.1) 168 (80.0)	**.001**
Tumor-related data			
Primitive cancer, n (%) Breast Gastrointestinal Kidney Liver Lung Lymphoma Melanoma Myeloma Prostate Sarcoma Thyroid Other Unknown	36 (11.1)* 14 (4.3) 31 (9.6) 1 (0.3) 112 (34.7)* 6 (1.9) 2 (0.6) 34 (10.5) 47 (14.6)* 6 (1.9) 2 (0.6) 28 (8.7) 4 (1.2)	61 (29.0) 7 (3.3) 26 (12.4) 2 (1.0) 52 (24.8) 3 (1.4) 2 (1.0) 13 (6.2) 16 (7.6) 3 (1.4) 4 (1.9) 18 (8.6) 3 (1.4)	** ** **.001** **.016** **.019**
Cancer Revealing metastasis Yes No	137 (42.4) 186 (57.6)	48 (28.9) 162 (77.1)	**.001**
Cancer history months (SD)	30.5 (33.4)	55.9 (56.1)	**.001**
Metastasis Free Survival, months (SD)	17.6 (1.6)	42.8 (3.7)	**.001**
Spine metastasis already known, n (%) Yes No	102 (31.6) 221 (68.4)	47 (22.4) 163 (77.6)	.023
Prior surgeon solicitation, n (%) Yes No	27 (8.4) 296 (91.6)	42 (20.0) 168 (80.0)	.001
Other organ metastases Yes No	115 (35.6) 208 (64.4)	83 (39.5) 127 (60.5)	.595
Number of affected levels, n (%) 1 2 3 >3	176 (54.5) 77 (23.8) 38 (11.8) 32 (9.9)	128 (61.0) 38 (18.1) 25 (11.9) 19 (9.0)	.394
Level Upper Cervical Lower Cervical Upper Thoracic Lower Thoracic Lumbo-sacral	4 (1.2) 32 (9.9) 130 (40.2) 91 (28.2) 66 (20.4)	4 (1.9) 25 (11.9) 47 (22.4) 50 (23.8) 85 (40.0)	**.001**
Circumferential topography Ant Post 360° (both)	39 (12.1) 13 (4.0) 271 (83.9)	33 (15.7) 17 (8.1) 160 (76.2)	.051
Tumor tissue invading the spinal canal Yes No	275 (85.1) 48 (14.9)	153 (72.9) 57 (27.1)	**.001**
Tumor tissue invading extra-spinal neighboring organs Yes No	53 (16.4) 270 (83.6)	30 (14.3) 180 (85.7)	.543
Tokuhashi, mean (SD)	8.8 (2.4)	10.0 (2.6)	**.001**

Values in bold are statistically significant.

### 3.2 Emergency referral pathway for SM

The referral patterns differed significantly between the groups; patients in the UGp were referred less often by oncologists than those in the EGp (39.0% vs. 58.6%, p<0.001). Patients in the UGp accounted for almost all referrals via emergency departments (68 vs. 1 patient); however, this mode of referral accounted for only 21.05% of the recruitment of the UGp. No significant difference was observed between the two groups in terms of referral by general practitioners (16.4% vs. 22.4%, p=0.1) or other specialists (23.5% vs. 18.6%, p=0.2).

**Figure 1:**
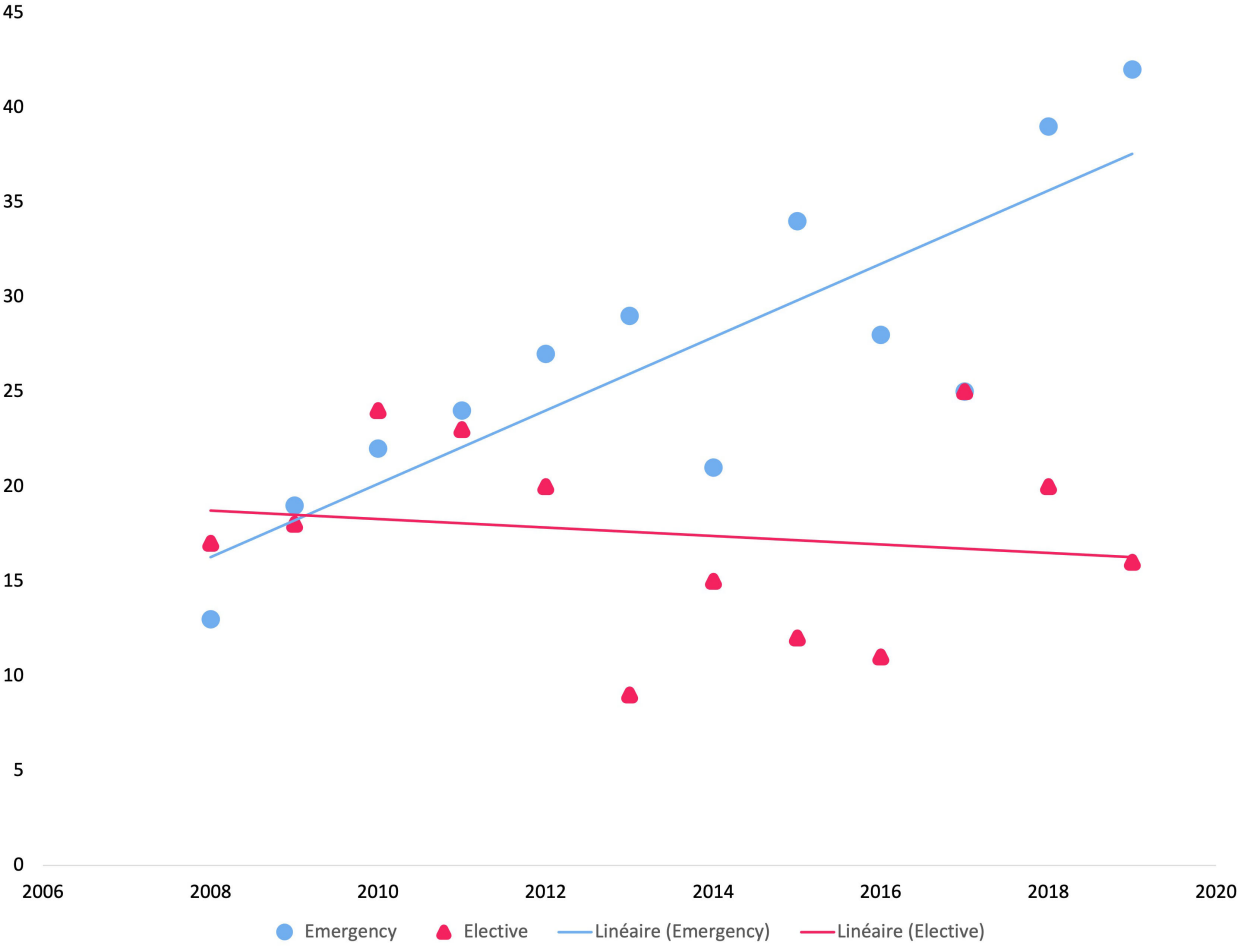
Evolution of referrals of patients with spinal metastases to surgery between 2008 and 2019 (n=533).

The percentage of patients referred for emergency surgery for SM (r^2 =^ 0.7) increased significantly over the 12-year period compared with that of those who were electively referred (r^2 =^ 0.02, p=0.005) (**Figure 1**).

**Figure 2:**
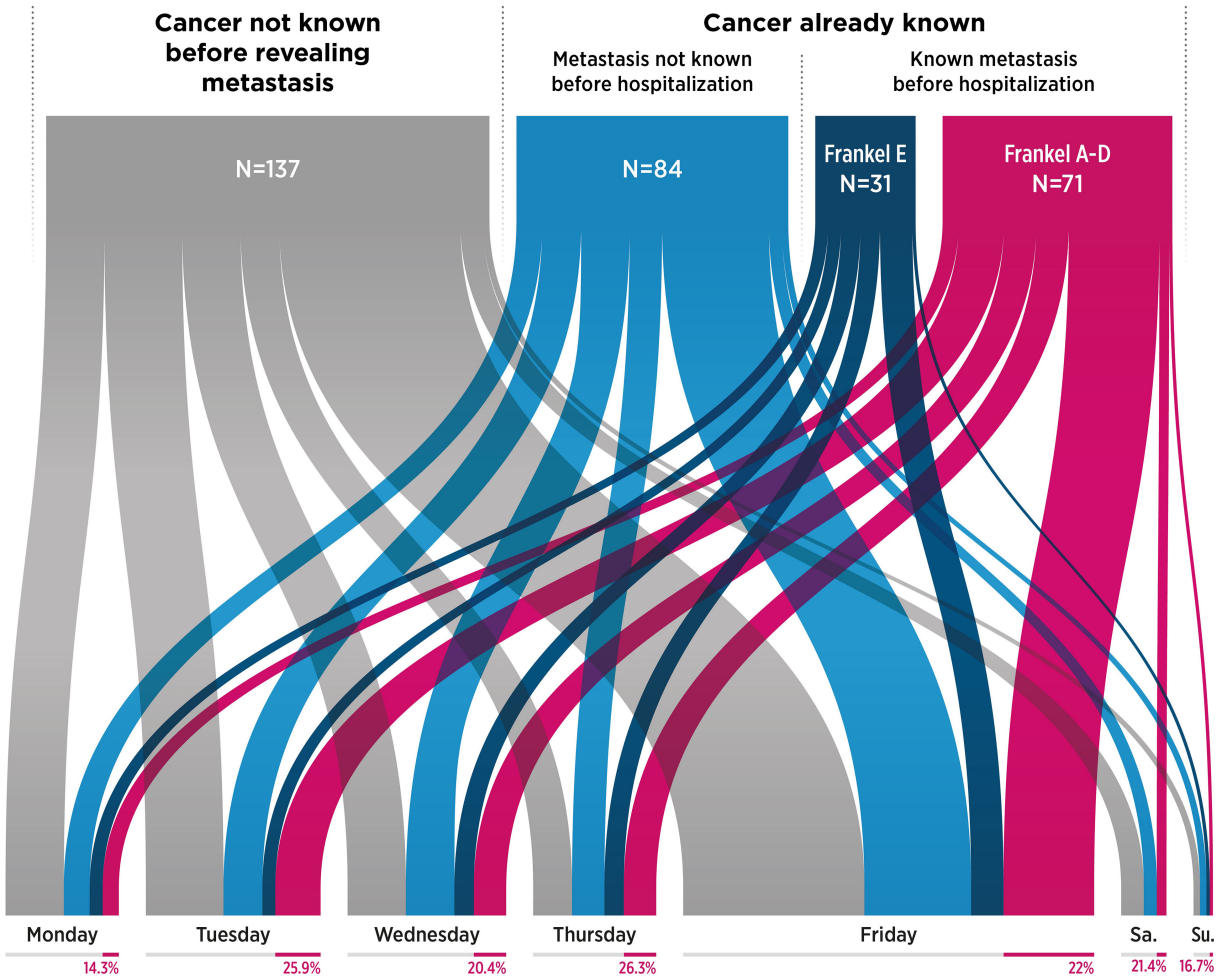
Distribution of patients with spinal metastases referred as emergency cases, according to oncological history, neurological status, and days of referral (UGp, n=323). (Frankel A-D: Presence of motor impairment; Frankel E: No motor impairment. The percentage under each day of the week indicates the number of patients treated as emergency cases with a known metastasis and motor deficit). UGp, urgent group.

Significantly more emergencies occurred on Friday than on other weekdays (39.3% vs. 1.9%–16.4%, p<0.0001), and this did not change throughout the study period. Only 6.2% of referrals occurred on Saturdays and Sundays combined (**Figure 2**).

### 3.3 Timeline of metastasis

Overall, 185 patients (34.7%) had metastases revealing cancer, 199 (37.3%) had metastases during the natural course of their monitored cancer, and 149 (27.9%) had already known metastasis.

More metastases were observed in the UGp than in the EGp (42.5% vs. 22.8%, p<0.0001).

**Figure 3:**
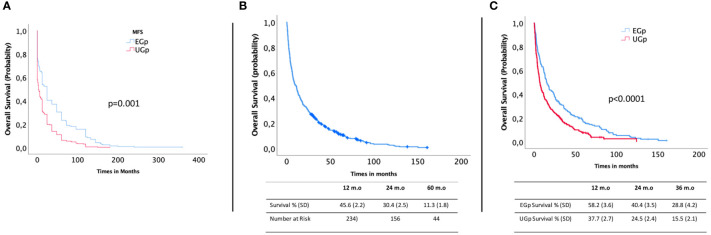
Metastasis-free survival for whole series **(A)**, overall survival for whole series **(B)**, and overall survival for the elective group (EGp, n=210) compared to that of the emergency group (UGp, n=323) **(C)**.

MFS was lower in the UGp than in the EGp (median 24.0 vs. 3 months, p<0.001, **Figure 3A**). The MFS was 0 months for metastases revealing cancer and 24 months for metastases in the natural course of cancer and already known metastases.

In the UGp, 186 (57.5%) patients had primitive cancer already monitored and 102 (31.6%) had already known SM (p<0.0001). On admission, 71 (69.9%) of the 102 patients presented with neurological deficits (Frankel A–D, **Figure 2**), and 27 of these 102 patients (26.4%) had previously received an upstream surgical opinion concluding that they should not be operated on, whereas 19 were treated as emergency cases with neurological impairment (Frankel A-D).

In the UGp, 125 (38.6%) patients had initial neurological symptoms that evolved over a few days, 164 (50.7%) for several weeks, and 34 (10.5%) for several months (p<0.0001).

### 3.4 Surgical outcomes

The onset of symptoms revealing metastases was more recent in the UGp than in the EGp (10.6 vs. 17.6 days, p<0.0001), and the patients in the UGp underwent surgery more rapidly after first contact with the surgeon (2.7 vs. 10.5 days, p<0.0001) (**Table 2**). Patients in the UGp were more likely to undergo a single decompression without fixation (54.8% vs. 34.3%, p<0.001). Patients in the UGp had a longer hospital stay (12.7 vs. 7.9 days, p<0.0001), but the perioperative complications did not differ (p=0.181). Evolution at the 3-month follow-up was significantly worse for the UGp (very poor and poor, 29.2% vs. 13.8%, p <0.0001).

**Table 2:** Differences in surgical and postoperative parameters between emergency (n=323) and elective patients with spinal metastases (n=210).

	Emergency (n=323)	Elective (n=210)	p value
Delay between surgeon first contact and surgery, days (SD)	2.7 (3.6)	10.5 (9.1)	**<.001**
Urgent surgery Yes No	226 (70.0) 97 (30.0)	12 (5.7) 198 (94.3)	<.001
Surgical procedure Decompression pure Any implants	177 (54.8) 146 (45.2)	72 (34.3) 138 (65.7)	<.001
Length of Stay, days (SD)	11.4 (9.4)	8.1 (5.2)	<.001
Complication Grade 0 Minor Major	266 (82.4) 19 (5.8) 38 (11.8)	184 (87.6) 8 (3.8) 18 (8.6)	.081
Evolution at 3 months Very good Good Fair Poor Very poor	6 (1.9) 143 (44.3) 80 (24.8) 47 (14.6) 47 (14.6)	9 (4.3) 136 (64.8) 36 (17.1) 15 (7.1) 14 (6.7)	<.001

Values in bold are statistically significant.

### 3.5 Risk factors for emergency surgical management

Using univariate and multivariate analyses, we identified risk factors for SM in patients who underwent emergency surgery (**Table 3**). Cox multivariate proportional hazards models identified short delay between onset of symptoms and first contact with a surgeon (<1 week) (OR 4.47, 95% CI 1.995–10.037, p<0.0001), initial motor deficit (OR 2.112, 95% CI 1.112–3.682, p=0.008), and non-ambulatory status (OR 8.36, 95% CI 3.178–17.819, p=0.002) as prognostic factors of undergoing emergency surgery. In contrast, SM diagnosis during cancer follow-up (OR 0.248, 95% CI, 0.131–0.470, p<0.0001) was associated with fewer emergency surgeries.

**Table 3:** Univariate and multivariate analysis for identifying risk factors of emergency surgery for spinal metastasis.

	Univariate analysis (p)	Multivariate analysis (p)	OR (CI 95%)
Primitive			
Breast	<.0001	**.007**	**0.43 (0.231–0.798)**
Lung	.0016	.589	1.174 (0.667–2.064)
Prostate	.0019	.556	1.275 (0.568–2.861)
Revealing spine Metastasis (Yes)	<.0001	**<.0001**	**4.28 (2.410–7.608)**
Known spine metastasis (Yes)	<.0001	**<.0001**	**3.78 (2.120–6.748)**
Epidural spreading	.001	.826	1.067 (0.600–1.895)
Vertebral level			
Cervical			
Thoracic	<.0001	.987	2.78 (0.002–12.82)
Lumbar	<.0001	**.009**	**0.45 (0.253–0.819)**
Precontact delay (between onset of symptoms and first contact with the surgeon)			
Days	<.0001	**<.0001**	**4.47 (1.995 –10.037)**
Weeks			
Months			
Frankel Classification			
Ambulatory (E-D)			
Non-ambulatory	<.0001	**.002**	**8.36 (3.178 –17.819)**

p-value was calculated using the log-rank test for univariate analysis and Cox regression for multivariate analysis. Values in bold are statistically significant.

### 3.6 Emergency and overall survival

The median OS was 9.86 months (range 7.7–12.0). The 12-, 24-, and 60-month OS estimates were 45.6% (SD 2.2), 30.4% (SD 2.5), and 11.3% (SD 1.8), respectively (**Figure 3B**). The median OS for the UGp was significantly lower at 12 months (37.7% vs. 58.2%), 24 months (24.5% vs. 40.4%), and 36 months (15.5% vs. 28.8%) (p<0.0001) (**Figure 3C**). The prognostic factors associated with the OS are presented in **Table 4**. In the multivariate proportional hazards model, we identified lung cancer (OR 1.420, 95% CI 1.134–1.6778, p=0.002), initial motor deficit (OR 1.485, 95% CI 1.200–1.837, p=0.001), emergency surgery (OR 1.384, 95% CI 1.108–1.729, p=0.0004), non-ambulatory status (Frankel A–C) (OR 2.294, 95% CI 2.413–6.048, p=0.041), major postoperative complications (OR 2.350, 95% CI 1.412–3.911, p=0.001), and short delay between the onset of symptoms and first contact with a surgeon (OR 1.260, 95% CI 1.060–1.497, p=0.009) as independent prognostic factors of poor OS. In contrast, primary breast tumors (OR 0.557, 95% CI 0.425–0.731; p<0.0001) and hemopathy (OR 0.446, 95% CI 0.319–0.624; p<0.0001) were associated with better OS.

**Table 4:** Univariate and multivariate analyses for survival prognosis factors.

	OS (months)	Univariate	Multivariable OR	pvalue
Sex		<0.0001		
Man	6.7 (0.7)		1.061 [0.824-1.367]	0.645
Women	16.2 (1.8)		–	
Primitive		<0.0001		
Breast	24.5 (4.3)		**0.557 [0.425-0.731]**	**0.026**
Lung	4.2 (0.7)		**1.420 [1.134-1.677]**	**0.002**
Hemopathy	35.3 (10.9)		**0.446 [0.319-0.624]**	**0.030**
Symptoms		<0.0001		
Motor	5.7 (0.6)		**1.485 [1.200-1.837]**	**0.001**
Pain	14.8 (1.4)		–	
Precontact delay (between onset of symptoms and first contact with the surgeon)		<0.0001		
Days	5.9 (0.7)		**1.260 [1.060-1.497]**	**0.009**
Weeks	11.5 (1.7)		0.855 [0.730-1.001]	0.056
Months	15.1(2.3)		–	–
Complications		<0.0001		
Minor	11.7 (1.3)		1.140 [0.736-1.768]	0.557
Major	1.8 (0.5)		**2.350 [1.412-3.911]**	**0.001**
KPS		<0.0001		
Good	13.0 (0.1)		0.715 [0.167-3.049]	0.650
Moderate	4.5 (0.6)		0.899 [0.214-3.776]	0.885
Poor	3.4 (0.9)		–	
Frankel Status		<0.0001		
Ambulatory (D-E)	12.4 (1.3)		0.847 [0.573-1.252]	0.404
No Ambulatory (A-C)	4.8 (0.8)		**2.294 [2.413-6.048]**	**0.041**
Other organ metastases	6.3 (0.6)	<0.0001	**1.541 [1.271-1.867]**	**0.024**
Known Spinal Metastasis	7.0 (0.9)	0.025	1.829 [0.769-1.719]	0.237
Emergency	6.740 (0.7)	<0.0001	**1.384 [1.108-1.729]**	**0.004**

p-value was calculated using the log-rank test for univariate analysis and Cox regression for multivariate analysis. Values in bold are statistically significant.

## 4 Discussion

### 4.1 A long-standing and unresolved issue

The management of threatening SM remains challenging, particularly with regard to timely referral to a spinal surgeon (25–28), which is a strong predictor of poor postoperative outcomes in symptomatic SM cases (29). Additionally, in 2013, the recommendations of the Third National Cancer Plan were published in our country (30), setting out specific objectives for all oncology stakeholders, including: (i) assessing the functioning of audits and quality of the tumor board and (ii) reducing inequalities in treatment delays by identifying barriers to adequate timelines. These objectives address the multidisciplinary problem of vertebral metastases, and despite significant budget allocation and regional reorganization, there is room for optimization in patient management.

This study highlighted dysfunctions in the management of patients with SM, symbolized by the increase in the number of patients admitted in emergency situations and the number of patients referred to emergency departments with already known metastases and neurological deficits. This deficit can be avoided by a better upstream organization (18, 25, 27, 31) that prioritizes the education of the patient, relatives, and medical teams and involves a surgeon earlier in the oncological process (32).

### 4.2 Impact of non‐elective management for SM

Reducing emergency referrals is one of the main goals of oncology research. Indeed, patients managed in emergency are significantly more likely to have neurological deficits than do elective patients (38.4% vs. 20% in our study), and several studies have reported a direct correlation between neurological deficits and reduced postoperative outcomes, quality of life, and survival (25, 33, 34). In fact, these results justify the need for rapid surgical intervention; the 48-hour cut-off point from symptom onset, which allows for better neurological recovery, defines a generally accepted threshold for emergency surgery (10, 11, 26). This need to operate quickly may conflict with the functioning of care units, especially on weekends or after-hours (35, 36).

### 4.3 The paramount values of symptoms

Emergency management of symptomatic SM involves decisions that could be of better quality if taken in advance within a multidisciplinary oncology pathway (31). Indeed, for patients with cancer with or without metastasis to be managed before the onset of a neurological deficit, patient and caregiver education should be optimized (18, 32). The average delay between the onset of any painful symptom and the onset of neurological deficits was found to be 7 weeks (37), prompting Levack et al. to advocate optimizing early radio-clinical assessment, so as not to wait for the onset of a neurological deficit (38). Although a neurological deficit may be the first symptom of cancer, most patients have a history of malignancy. Therefore, symptoms suggestive of neurological decompensation, such as atypical back pain aggravated by movement, radicular pain, or ataxia, may also be present. In our study, 50.7% of emergency patients experienced symptoms for several weeks and 10.5% experienced symptoms for several months. Husband et al. described a median total delay (from the onset of complaints to treatment) of 73.5 days (16), Levack et al. found a median total delay of 90 days (38), and VanTol et al. demonstrated a total delay of 99 days from the first symptoms to treatment (39). The need to optimize the delay in identifying symptoms, performing paraclinical examinations, and transferring the patient to a specialized environment are essential elements of management. The functional outcome of malignant spinal cord compression depends on functional status at the time of treatment (16, 40). Thus, the attention of correspondents, especially residents, must be refocused on the value of clinical examinations to encourage practitioners from all disciplines to recognize early signs and the need for prompt referral (11). This delay in management could be addressed by increasing physician, patient, and family awareness of the alarming symptoms. In most health systems, the general practitioner is pivotal in the coordination of care and must be involved in this clinical reactivity, because the discrimination of radicular pain or an early neurological deficit is not easy in the general context of cancer (32, 39, 41).

Finally, the involvement of patients in their self-assessment is key to detecting symptoms that may lead to consultation at an early stage. The development of a new technological approach involving patient-reported outcomes is promising. In the context of advanced or metastatic disease, measurement of these outcomes is valuable for detecting symptoms or functional impairment associated with both disease and treatment (42).

### 4.4 Improved multidisciplinarity

Notably, emergency admissions increased during the investigation period, and patients sometimes presented with urgent neurological deficits despite known metastases from a known cancer. SM may pose a problem in the organization of an oncology network. In 2000, Galasko et al. expressed concern that spinal surgeons had failed to educate their colleagues in other specialties about the principles of management of spinal instability secondary to metastatic spinal disease (18). This pessimistic observation was made before the advent of the SINS (3); however, Guzik et al. recently reported that spinal surgeons are not systemically integrated with tumor boards (31). The systematization of multidisciplinary meetings has been a great step forward for the management of patients with cancer (43, 44), but no decision can be made on spinal imaging without the expertise of a spinal surgeon (45).

When a board dedicated to SM is established, the number of patients managed in the emergency room decreases significantly over time (46). Our study (without a specific tumor board) illustrates the opposite, thus arguing in favor of this type of organization. For obvious reasons, the presence of a surgeon at all meetings is illusory in terms of planning, but technical innovations can be implemented to allow the surgeon to participate in decision-making, even in a delocalized manner (47). The potential of telemedicine, already applied to other “virtual” oncology meetings, is promising in the field of spinal metastatic pathology, including in emergencies (32, 48, 49).

Organizing a multidisciplinary network on a regional scale and integrating complex pathologies with multiple stakeholders and institutions are challenging (45). However, in light of our study, some simple key points could be communicated to all parties involved in the management of SM: the irreplaceable value of repeated neurological clinical examinations, development of early access to neuroimaging, creation of boards involving surgeons (possibly in telemedicine), integration of scores (particularly SINS) in the decision-making process, and implication of a systematic surgical opinion for any abnormal imaging finding (28, 46, 50, 51).

### 4.5 Ideal surgical timing: a question of common sense?

There is still considerable variability in the ideal timing between institutions and even between practitioners. The bone of contention remains that, in clinical practice, magnetic resonance imaging findings of spinal cord compression are not always correlated with the severity of paralysis. Although high-grade epidural spinal cord compression (ESCC) is often an indication for surgical decompression, there is no consensus in the literature on the precise definition of this term. Thus, some authors, such as Bilsky et al., assumed that the ESCC scale would be a valid and reliable instrument that could be used as a major determinant in the decision to operate or perform radiotherapy (52), while others, such as Uei et al., concluded that the severity of paralysis was not correlated with the ESCC scale (53).

Ideally, surgical decompression should be a matter of the shared experience of the surgeon, combined with that of the oncologists/radiotherapists and radiologists, depending on i) the aggressiveness and radiosensitivity of the primary tumor, ii) delay required to benefit from radiotherapy, and iii) radiological criteria of suffering or spinal cord injury. In our practice and in line with some of the recommendations mentioned above, we strongly emphasize the need to perform at least one decompression for high-grade epidural compression by non-highly radiosensitive tumors, even if the patient still has no neurological deficits (54–56).

However, the indication for stabilization surgery is no longer debated and remains guided by the Spine Instability Neoplastic Score (SINS) associated with mechanical pain (55, 57). It incorporates radiological criteria for predicting spinal stability in patients with neoplastic lesions. A meta-analysis by Pennington et al. showed good intra- and inter-observer reliability of the SINS system for the diagnosis of mechanical instability, and it appears that systematic use of this scoring system in clinical practice has led to a reduction in spinal instability in radiotherapy and surgery cohorts through earlier surgical referral of patients (58, 59). In our view, the challenge remains to integrate these tools into teams that are open to systematizing them in daily practice.

### 4.6 Biases and limitations

This study had some limitations because it was a non-randomized retrospective study with no data on postoperative health-related quality of life. In addition, the criteria guiding surgical decisions varied. Unfortunately, we could not fully analyze the causes of surgical referral delays, as did Van Tol et al. (39), because information on the organizational aspect of other institutions upstream was almost impossible to obtain objectively. In addition, a record of the degree of epidural compression at the time of diagnosis, as well as the SINS instability score, would have been of interest to highlight and determine the optimal timing for operation in patients. Over the course of our study, we found that these data were not integrated or used daily by various stakeholders in the oncology network.

## 5 Conclusion

Our study confirmed a dysfunction in the global management of SM, leading to a delay in the referral to spinal surgery. The outcome of patients managed in an emergency is unfavorable compared to that of elective patients, and the rate of urgent referrals must be reduced, especially among those with already identified metastases.

This multi-causal situation persists in a health system that has made cancer a major national priority and has devoted significant resources to optimizing multidisciplinary care and reducing delays in access to care.

All stakeholders managing these patients with cancer must exhibit a proactive attitude, recognize the symptoms that may lead to the suspicion of spinal metastatic localization, and work in a synergistic way to limit the delays in referral to surgery and inherent emergency management.

## Data availability statement

The raw data supporting the conclusions of this article will be made available by the authors, without undue reservation.

## Ethics statement

This study was approved by the Ethics Committee of the College of Neurosurgery (n° IRB00011687) IRB #1: 2022/08. The research was conducted in accordance with local legislation and institutional requirements. Written informed consent for participation was not required from participants due to retrospective database analysis.

## Author contributions

BD: Conceptualization, Data curation, Formal Analysis, Investigation, Methodology, Project administration, Visualization, Writing – original draft, Writing – review & editing. AP: Conceptualization, Investigation, Methodology, Project administration, Writing – original draft. GL: Conceptualization, Data curation, Supervision, Writing – review & editing. OH: Formal Analysis, Investigation, Supervision, Validation, Writing – review & editing. JD: Formal Analysis, Investigation, Supervision, Validation, Writing – review & editing. MD: Conceptualization, Data curation, Supervision, Writing – review & editing. GD: Supervision, Writing – review & editing. CB: Supervision, Writing – review & editing. IL: Formal Analysis, Supervision, Validation, Writing – review & editing. AA: Conceptualization, Data curation, Investigation, Resources, Supervision, Writing – review & editing.
